# ROHHAD syndrome and evolution of sleep disordered breathing

**DOI:** 10.1186/s13023-016-0484-1

**Published:** 2016-07-30

**Authors:** Diana Reppucci, Jill Hamilton, E Ann Yeh, Sherri Katz, Suhail Al-Saleh, Indra Narang

**Affiliations:** 1 Hospital For Sick Children, Toronto, Ontario Canada; 2 University of Toronto, Ontario, Canada; 3 Children’s Hospital of Eastern Ontario and University of Ottawa, Ontario, Canada; 4 Division of Respiratory Medicine, Hospital for Sick Children, 555 University Ave, Toronto, ON M5G 1X8 Canada

**Keywords:** ROHHAD syndrome, Sleep disordered breathing, Nocturnal hypoventilation, Polysomnogram

## Abstract

**Background:**

Rapid-onset obesity with hypothalamic dysfunction, hypoventilation and autonomic dysregulation (ROHHAD) is a rare disease with a high mortality rate. Although nocturnal hypoventilation (NH) is central to ROHHAD, the evolution of sleep disordered breathing (SDB) is not well studied. The aim of the study was to assess early manifestations of SDB and their evolution in ROHHAD syndrome.

**Methods:**

Retrospective study of children with ROHHAD at two Canadian centers. All children with suspected ROHHAD at presentation underwent polysomnography (PSG) to screen for nocturnal hypoventilation. PSG findings at baseline and follow-up were collected. Interventions and diagnostic test results were recorded.

**Results:**

Six children were included. The median age of rapid onset obesity and nocturnal hypoventilation (NH) was 3.5 and 7.2 years respectively. On initial screening for ROHHAD 4/6 (66.7 %) children had obstructive sleep apnea (OSA), 1/6 (16.7 %) had NH and 1/6 (16.7 %) had both OSA and NH. Follow up PSGs were performed in 5/6 children as one child died following a cardiorespiratory arrest. All children at follow up had NH and required non-invasive positive pressure ventilation. Additionally, 3/6 (50 %) children demonstrated irregular breathing patterns during wakefulness.

**Conclusions:**

Children with ROHHAD may initially present with OSA and only develop NH later as well as dysregulation of breathing during wakefulness. The recognition of the spectrum of respiratory abnormalities at presentation and over time may be important in raising the index of suspicion of ROHHAD. Early recognition and targeted therapeutic interventions may limit morbidity and mortality associated with ROHHAD.

## Background

Rapid-onset obesity with hypothalamic dysfunction, hypoventilation and autonomic dysregulation (ROHHAD) is a rare, heterogeneous syndrome and responsible for hypothalamic obesity. So far more than 75 cases have been reported since 1965 when it was first described in the literature. No specific diagnostic test has been established yet, and the natural history of the condition remains poorly understood [1]. The most common early symptom reported in ROHHAD is excessive weight gain (20–30 pounds over 6–12 months) in a young child beginning at the age of 2–3 years [2, 3]. Additional features of ROHHAD include neuroendocrine tumor, hyperprolactinemia, central hypothyroidism, disordered water balance, failed growth hormone stimulation test, temperature dysregulation and hypotension. However, given the lack of standardized definitions and diagnostic tests as well as epidemiological studies of ROHHAD, the evolution and spectrum of abnormalities is unclear [2, 3]. Nonetheless, a high burden of morbidity, as well as mortality rates between 50 to 60 % necessitates early diagnosis and subsequent management of ROHHAD, which may lead to a decrease in morbidity and mortality [2, 3].

Previous published case reports and reviews have described obstructive sleep apnea (OSA), central sleep apnea (CSA) and abnormal ventilatory responses to carbon dioxide (CO_2_) co-existing with nocturnal hypoventilation (NH). Specific data on sleep disordered breathing (SDB) at presentation and its evolution in children with suspected ROHHAD are limited [3–7]. As SDB in children with ROHHAD may predispose to later cardiorespiratory arrest, studying the evolution of SDB in ROHHAD may be crucial for the early recognition and targeted treatments in ROHHAD.

We hypothesized that children with ROHHAD may not always present with NH at an early stage of ‘ROHHAD disease’ and NH may evolve over time. The aim of this case series was to review the baseline and follow up polysomnograms in children with suspected ROHHAD and to describe the evolution of their SDB over time.

## Methods

We conducted a retrospective case series review in children with ROHHAD at two pediatric institutions across Canada (The Hospital for Sick Children, Toronto and the Children’s Hospital of Eastern Ontario, Ottawa). All sleep history data and PSG findings were accessible through the sleep charts and physician’s reports. We reviewed all demographic data, age at onset of obesity, symptoms at presentation and age at onset of nocturnal hypoventilation (NH). All symptoms related to hypothalamic and autonomic dysfunction were also recorded.

Additional information such as diagnostic tests and blood parameters were also obtained. We included all children diagnosed with ROHHAD between 0 and 18 years of age. The criteria for the diagnosis of ROHHAD which is described in its acronym, include rapid onset obesity, hypothalamic dysfunction, nocturnal hypoventilation and autonomic dysregulation. The absence of PHOX2B mutation helps to rule out Congenital Central Hypoventilation Syndrome [2–4]. For the purpose of this study, the above mentioned defintion of ROHHAD was adapted and defined if the following 7 criteria were met: 1. history of rapid onset obesity, 2. hypothalamic dysfunction, 3. autonomic dysregulation, 4. sleep breathing disorders (including nocturnal hypoventilation and/or obstructive sleep apnea and/or central apnea as defined by a formal overnight PSG described below), 5. a negative test for PHOX2b mutation, 6. a normal brain MRI scan and 7. the absence of any genetic mutation that may account for obesity, autonomic or hypothalamic dysfunction.

### Polysomnography

Children with suspected ROHHAD were sent to our sleep clinic for a PSG to confirm the diagnosis by the presence of NH. Hence, all our initial PSG were done when the clinical diagnosis was still uncertain. All children underwent standard overnight PSG using a Natus Sleepworks system (Natus Medical Incorporated, San Carlos, California, United States) according to standard international guidelines [8].

PSG measurements included electroencephalogram, electro-oculogram, submental and bilateral anterior tibialis electromyogram. Respiratory measurements included chest wall and abdominal belts; nasal air pressure transducer and/or oronasal thermal sensor, oxygen saturation, end-tidal and/or trans-cutaneous carbon dioxide monitors. Video and audio recording as well as body position were recorded.

Sleep architecture was assessed by standard techniques. PSG recordings were made according to the standards of American Academy of Sleep Medicine (AASM) [8]. Recorded respiratory data included counts and indices of the following events: obstructive apnea, central apnea, hypopnea and mixed apneas in non-rapid eye movement (NREM) sleep, REM sleep and total sleep. All respiratory events were scored according to the AASM scoring guidelines by a registered polysomnographic technician [8]. OSA severity was graded according to OAHI, the number of obstructive apneas, obstructive hypopneas and mixed apneas per hour during sleep. OAHI of < 1.5 was considered normal, OAHI from ≥ 1.5 to < 5 was mild OSA; OAHI from ≥ 5 to < 10 was moderate OSA and OAHI ≥ 10 was considered severe OSA. The central apnea index (CAI) was defined as the number of central apneas per hour during sleep. A CAI ≥ 5 per hour was considered significant. The mean and nadir nocturnal oxygen saturation (SaO_2_) was recorded for each patient from the overnight PSG as well as the mean and peak nocturnal CO_2_. Definition of nocturnal hypoventilation (AH) was used in accordance to AASM 2007 as a CO_2_ > 50 mmHg for more than 25 % of the total sleep time (TST). Specific management or intervention including adenoidectomy, tonsillectomy, and initiation of non-invasive positive pressure ventilation (NIPPV) such as continuous positive airway pressure (CPAP) or Bi-level PAP (Bi-level) following the PSG findings were also recorded.

In addition, three of six children underwent daytime cardiorespiratory monitoring to evaluate daytime cardiopulmonary physiology. Specifically, this involved continuous measurements of heart rate, respiratory rate, oxygen saturations, transcutaneous CO_2,_ respiratory and abdominal excursions using chest wall and abdominal belts as well as the use of a nasal airflow channel. This was undertaken in the sleep laboratory setting while seated upright and awake in a chair for the entire duration. The subject was allowed to read or watch television during this time. None of the patients fell asleep during this monitoring period. Given that there are no defined criteria for scoring central pauses while awake, central pauses were scored in accordance to AASM. A central apnea was recorded if they met the following criteria: the event lasts 20 s or longer or it lasts at least the duration of two breaths, is associated with a 3 % or greater oxygen desaturation [8]. No EEG data was applied during wakefulness.

### Statistical analyses

The Statistical Package for Social Sciences software (SPSS 20.0) was used for the calculations of all the parameters. Baseline characteristics (sex, onset age of obesity, onset age of nocturnal hypoventilation, weight and BMI) and sleep study variables were evaluated by calculating the proportions, median and range.

## Results

**Fig. 1:**
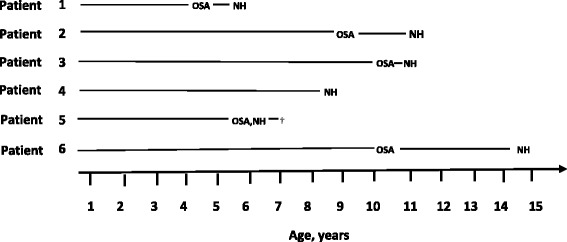
Sequence of Sleep Disordered Breathing Phenotype in each patient. Abbreviations: OSA Obstructive Sleep Apnea, NH Nocturnal Hypoventilation, † Patient died. Age of presentation for SDB is shown on the x-axis for each patient

We retrospectively identified six children with ROHHAD. All demographic data are summarized in Table 1. PSG findings at baseline and follow up are listed in Table 2. The majority of children (83.3 %) presented with rapid weight gain during early childhood, while one child presented later at 9.5 years of age. The sequence of SDB phenotypes of each patient is shown in Fig. 1. NH which is reported to be a cardinal feature of ROHHAD, was diagnosed at a median age of 7.2 years. Additional features at presentation in children being evaluated for suspected ROHHAD syndrome included hypothalamic dysfunction, adipsia, diaphoresis, heat intolerance, behavioral problems and cardiorespiratory arrest.

**Table 1:** Demographic Data of patients with ROHHAD syndrome

Characteristics	Results
	*n* = 6
Male, number (%)	1 (16.7)
Age of onset of obesity (years)	3.5 (1.5–9.5)
Age of onset of nocturnal hypoventilation (years)	7.2 (5.3–14.7)
BMI at baseline PSG (kg/m^2^)	32.1 (26.5–40.7)

All values are median (range) unless stated otherwise

**Table 2:** Baseline and follow up PSG data in children with suspected ROHHAD

Baseline PSG							Follow-up PSG				
Pt	BMI (kg/m^2^)	Age (years)	PSG Diagnosis	SaO_2_ Min (%)	Co2 range (mmHg)	Treatment	• Time interval (years) • N of PSG	BMI (kg/m^2^)	PSG diagnosis	CO_2_ range (mmHg)	Treatment
1	35.7	4.7	Mild OSA	79	41–47	Weight loss	• 1y 4 m	43.5	Severe OSA and NH	50–54	• Bi-Level PAP
			• OAHI 3/h • CAI 0/h				• 5		• OAHI 12/h • CAI 0/h		• Suppl. Oxygen (day)
2	28.5	9.0	Mild OSA	82	41–49	Weight loss	• 2y 5 m	36.4	Moderate OSA and NH	35–52	Bi-Level PAP
			• OAHI 4/h • CAI 0/h				• 3		• OAHI 9/h • CAI 0/h		
3	26.5	10.1	Severe OSA	83	29–38	•Adenotonsillectomy • Weight loss	• 6 m	26.6	Severe OSA and NH	40–53	• Bi-Level PAP
			• OAHI 49/h • CAI 0/h				• 1		• OAHI 142/h • CAI 2.9/h		• Oxygen (day and night)
4	40.7	8.3	NH	90	47–60	Bi-Level PAP	• 8 m	40.4	No change	#32–39	Maintained on Bi-Level PAP
			• OAHI 0/h • CAI 0/h				• 1		• OAHI 0/h • CAI 0/h		
5	40.8	5.3	Severe OSA and NH	67	50–86	Bi-Level PAP	-	-	• NOT PERFORMED	-	-
			• OAHI 11/h • CAI 0/h						• Pt deceased		
6	39.9	10.0	Mild OSA	81	38–44	Weight loss	• 4y 8 m	47.6	Severe OSA and NH	Peak 51	Bi-Level PAP
			• OAHI 4/h • CAI 0/h				• 8		• OAHI 18/h • CAI 0/h		

Time Interval describes the time between the first and follow-up PSG in the table; Number of PSG refers to the total number of PSG that were performed between the first PSG and the follow-up PSG in the table; # - patient number 4 was not hypercapnic on follow-up PSG as this was undertaken on Bi-Level PAP

Abbreviations: *OSA* obstructive sleep apnea, *NH* nocturnal hypoventilation, *OAHI* obstructive apnea hypopnea index, *CAI* central apnea index, *AT* adenotonsillectomy

At baseline 2/6 (33.3 %) children demonstrated NH, 4/6 (66.7 %) children had OSA ranging from mild to severe. The highest CO_2_ parameter was 86 mmHg and the lowest SaO_2_ was 67 % recorded in a 5 year old female with severe OSA co-existing with NH. Management following the baseline PSG consisted of the following: three children with mild OSA were encouraged to lose weight as the primary treatment, two children were started on Bi-Level PAP and one patient with severe OSA underwent an adenotonsillectomy as the first line of treatment.

Follow up PSGs were performed in 5/6 children as one child died prior to a repeat PSG. Follow up PSG were performed typically 1 year after Bi-level PAP initiation. Patient #3 who had severe OSA and underwent adenotonsillectomy showed NH 6 months after the first PSG. In contrast three patients (#1, # 2 and #6) who did not have NH in their first PSG required between 3 and 7 PSGs (conducted between biannually and yearly) until we documented NH. In addition, four children had evidence of NH only after repeat PSGs were performed. These four children had previously documented mild to severe OSA (Table 2).

### Daytime cardiorespiratory monitoring

**Fig. 2:**
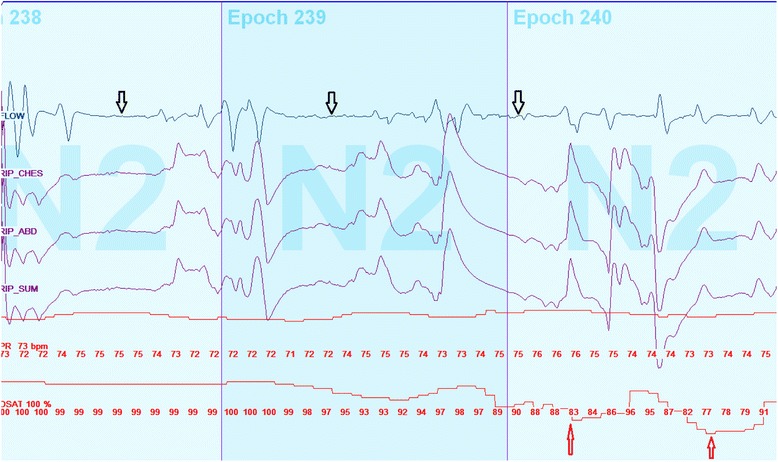
Cardiorespiratory monitoring during wakefulness (patient #3). Ten year old male with abnormal control of breathing during wakefulness. The test was performed while the child was sitting and watching tv. This was a 90 s recording during wakefulness which shows several central pauses (*black arrow*) with associated mild to moderate oxygen desaturations (*red arrow*). The desaturations were transient with recovery to baseline

**Fig. 3:**
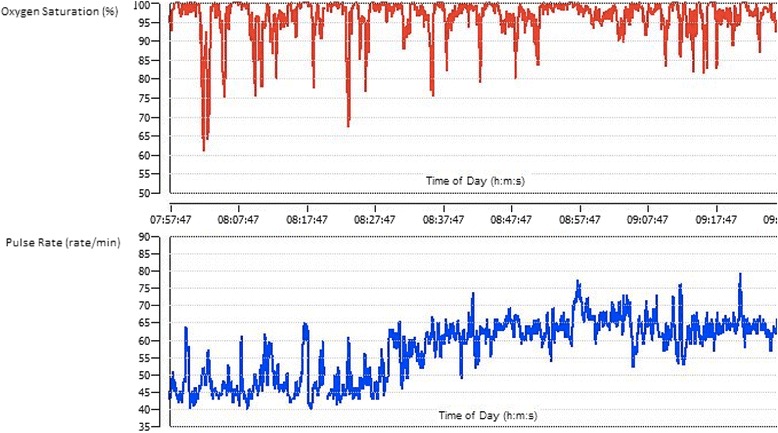
Oxygen saturations and heart rate during wakefulness (patient #3). Ten year old male with mild to severe oxygen desaturations and heart rate variability while awake. Desaturations to lowest SaO_2_ of 60 % and bradycardia of 40 beats per minute were recorded. These events were transient and returned to baseline without any need of intervention

Due to the cardiorespiratory arrest in one of our patients and evidence for of daytime oxygen desaturations during wakefulness, 3/6 (50 %) children underwent daytime cardiorespiratory monitoring. We were unable to perform this daytime monitoring in one patient who died and the remaining patients refused the testing due to parental work commitments. Cardiorespiratory variables during wakefulness are summarized in Table 3. All of these three children had central pauses while awake (see example in Fig. 2), durations of which ranged between of 10–47 s. There were mild to moderate oxygen desaturations (lowest recorded oxygen saturation while awake was 40 %) with a desaturation index between 7.5/h and 35/h. One of these three children (child #1) was diagnosed with pulmonary hypertension after. All three children were commenced on daytime oxygen therapy to keep their oxygen saturation above 94 %. No patient required NIPPV during wakefulness. In addition, child #3 had decreases in heart rate to 40/min during daytime monitoring (Fig. 3) but did not have persistent bradycardia of less than 40 beats per minute. Despite severe desaturations while awake in child #3 as well as significant OSA associated with nocturnal hypoventilation, the child and his family refused a tracheostomy and ventilation strategy.

**Table 3:** Cardiorespiratory variables during monitoring while awake

Pt	Age (years)	Heart rate average (rate/min)	Respiratory rate average (breaths /min)	tcCO_2_ range (mmHg)	Total time of study (min)	Desaturation index (/hr)	SaO_2_ nadir (%)
1	9.4	100	24	37–52	260	35	73
2	14.3	97	29	35–48	270	7.5	93
3	11.2	69	21	38–49	358	25.6	62

Daytime cardiorespiratory monitoring was performed while patients were sitting and watching tv. All patients who underwent the test showed abnormal control of breathing during wakefulness

Abbreviation: *tcCo2* transcutaneous CO2

Although the main aim of our study was to assess early manifestation and to document the evolution of SDB in children with suspected ROHHAD, other features of ROHHAD syndrome are shown in Table 4.

**Table 4:** Hypothalamic and autonomic features present in ROHHAD patients

Phenotype	Number
Hypothalamic Dysfunction	
Hyperprolactinemia	4
Hypothyroidism	4
Hypernatremia	3
Polydipsia	2
Hyperphagia	3
Growth Hormone Deficiency	2
Adipsia	1
Adrenal Insufficiency	1
Diabetes Insipidus	1
Autonomic Dysregulation	
Bradycardia	3
Thermal Dysregulation	3
Gastrointestinal Dysmotility	2
Hypotension	2
Tumor of neural crest origin	1

## Discussion

ROHHAD is a rare and fatal disease with a mortality rate of up to 50 to 60 % due to cardiorespiratory arrest. The diagnosis of ROHHAD can be extremely challenging as there is no single confirmatory diagnostic test, but the early recognition and intervention of this syndrome may minimize mortality [2, 9]. This study focused on SDB in children with ROHHAD syndrome, to ascertain how many children had NH at presentation, given that NH is defined as a cardinal feature of ROHHAD. This is important as excluding a diagnosis of ROHHAD of due to a lack of evidence of NH may result in catastrophic consequences.

In our study, only 2/6 patients had NH with it at baseline in our series. OSA was the most common SDB at presentation. Even though OSA has been documented in ROHHAD syndrome it’s not included in the diagnostic criteria. However, in our study, OSA was the initial presentation in regards of SDB. There was evidence of NH in all our patients but only over time. Moreover, we found that of the children studied, there was evidence for abnormal control of breathing during wakefulness with central pauses in breathing and associated oxygen desaturations.

Rapid onset obesity, a major presenting feature of ROHHAD syndrome was documented in all our children at a median age of 3.5 years, with one child presenting beyond 9 years of age, similar to other reported studies [2, 3, 7, 10]. The median age of onset of NH in our study was 7.2 years (range 5.3–14.7 years). In the largest case series thus far, Ize Ludlow and colleagues [2] report on the respiratory manifestations of 15 ROHHAD patients. All 15 patients had evidence for NH and 8/15 (53 %) had co-existent OSA and 4/15 (27 %) had cyanotic episodes. However, whether NH was present at diagnosis or evolved over time was not described. Alarmingly 9/15 (60 %) patients were reported to have had a cardiorespiratory arrest, much higher than in the current study (1/6, 17 %). Similarly in another case series of 13 patients with suspected ROHHAD, all 13 patients were reported as having NH and it is unclear if they had other pre-existing sleep related respiratory disorders [4].

The aforementioned case series and additional case reports offer a great deal of insight into the respiratory manifestations of ROHHAD syndrome [2, 5, 10–12].

Although NH is a definitive criteria of ROHHAD, children who fit all diagnostic criteria except NH in an overnight PSG need vigilant follow up with serial PSGs as NH may develop over time. As such, it is important to recognize that other sleep disorders may be associated with ROHHAD prior to catastrophic respiratory events.

Additional findings in our study showed evidence for hypoxemia during wakefulness, characterized in our study to be related to central pauses which may be an indicator of worse respiratory morbidity. Indeed, blunted chemosensory responses such as abnormal respiratory response to hypercapnia has been previously described in children with ROHHAD which may account for our observations [6]. These observations may be critically important in the context that other authors have suggested that early intervention with nocturnal artificial ventilation may improve daytime ventilation [13].

In our study 2/6 (33.3 %) patients were diagnosed with AH and treated with nocturnal Bi-level ventilation after the initial PSG. However, prior to a follow up PSG, one patient died following a cardiorespiratory arrest. The remaining 5/6 patients all required Bi-Level ventilation with 2/6 requiring supplemental oxygen during the day. In contrast to other studies, no patient required either invasive nor full time ventilatory support [2, 4, 5]. However, a child with significant daytime ventilatory abnormalities not controlled with oxygen therapy coupled with NH would likely require a tracheostomy and invasive ventilation but no patient in our series fit this criteria. Serial PSGs and repeated daytime monitoring will be necessary to ensure these patients are stable with their current ventilatory support. Interestingly, in a recent review of 51 cases of ROHHAD syndrome, 69 % required artificial ventilation via tracheostomy with 31 % requiring 24 h support which concur with data from other published case series [5]. The high incidence of invasive ventilation among those ROHHAD patients compared with our study may be related to more severe phenotype of ROHHAD associated with significant NH at a young age (mean age of invasive ventilation was 3.8 years). Furthermore, differences in clinical practice and use of Bi-level ventilation versus ventilation via a tracheostomy between countries could account for some of the variations observed in the mode of ventilation. Similar to the other published data, cardiorespiratory arrest is a manifestation of ROHHAD and one child in our study died from a sudden event [2–4].

The strength of our study was the inclusion of children with baseline PSGs before they were diagnosed with ROHHAD syndrome’. The limitations of our study include the retrospective design, lack of standardization with regards to timing of follow up PSG monitoring and the lack of a control group of similarly obese children to compare effects of a change in BMI that may have contributed to associated obesity-associated hypoventilation. Given the rarity of this condition, an additional, substantial limitation of our study, is the small number of patients included. Further, the daytime physiological monitoring could be performed only in 3/6 patients as 1 died and 2 were refused by the parents. Finally, we did not have pulmonary function tests on our patients to further explain abnormal gas exchange observed nocturnally. A larger multi centered study with children suspected of having ROHHAD is needed to confirm the findings of our study.

In summary, we have shown that children with suspected ROHHAD syndrome may not have NH at presentation but may only have evidence of mild OSA on their PSG. The absence of NH at presentation does not exclude ROHHAD, as NH may develop over time and thus only be evident with serial PSGs. Furthermore, we found evidence of abnormal breathing patterns during wakefulness with associated hypoxemia. Based on these findings, the authors would advocate for close and serial monitoring with PSGs of all children with suspected ROHHAD including daytime monitoring of cardiopulmonary variables once NH is evident. Importantly, the recognition of the spectrum of respiratory abnormalities associated with suspected ROHHAD syndrome at presentation and over time may be important in raising the index of suspicion for ROHHAD condition, and predispose to targeted interventions to limit associated morbidity and mortality.

## Conclusions

Children with suspected ROHHAD syndrome with no evidence of nocturnal hypoventilation on polysomnogram should be monitored closely by serial polysomnograms. A missed or delayed diagnosis of ROHHAD syndrome can lead to fatal consequences such as cardiorespiratory arrest.

## Abbreviations

AT, adenotonsillectomy; Bi-level PAP: Bi-level positive airway pressure; CAI, central apnea index; CO2, carbon dioxide; CSA, central sleep apnea; NH, nocturnal hypoventilation; NIPPV, non-invasive positive pressure ventilation; NREM, non rapid eye movement; OAHI, obstructive apnea hypopnea index; OSA, obstructive sleep apnea; REM, rapid eye movement; ROHHAD, rapid-onset obesity with hypothalamic dysfunction, hypoventilation and autonomic dysfunction; SDB, sleep disordered breathing; tcCO2, transcutaneous carbon dioxide
